# A systematic review and meta-analysis of the prevalence of poor sleep in inflammatory bowel disease

**DOI:** 10.1093/sleepadvances/zpac025

**Published:** 2022-08-26

**Authors:** Alex Barnes, Réme Mountifield, Justin Baker, Paul Spizzo, Peter Bampton, Jane M Andrews, Robert J Fraser, Sutapa Mukherjee

**Affiliations:** Department of Gastroenterology, Southern Adelaide Local Health Network (SALHN) Flinders Medical Centre, Bedford Park South Australia, Australia; Adelaide Institute for Sleep Health, Flinders Health and Medical Research Institute, College of Medicine and Public Health, Flinders University, Bedford Park, South Australia, Australia; Department of Gastroenterology, Southern Adelaide Local Health Network (SALHN) Flinders Medical Centre, Bedford Park South Australia, Australia; Adelaide Institute for Sleep Health, Flinders Health and Medical Research Institute, College of Medicine and Public Health, Flinders University, Bedford Park, South Australia, Australia; Department of Gastroenterology, Southern Adelaide Local Health Network (SALHN) Flinders Medical Centre, Bedford Park South Australia, Australia; Adelaide Institute for Sleep Health, Flinders Health and Medical Research Institute, College of Medicine and Public Health, Flinders University, Bedford Park, South Australia, Australia; Department of Gastroenterology, Southern Adelaide Local Health Network (SALHN) Flinders Medical Centre, Bedford Park South Australia, Australia; Adelaide Institute for Sleep Health, Flinders Health and Medical Research Institute, College of Medicine and Public Health, Flinders University, Bedford Park, South Australia, Australia; Inflammatory Bowel Disease Service, Department of Gastroenterology and Hepatology, (CAHLN) Royal Adelaide Hospital, Adelaide, South Australia, Australia; School of Medicine, Faculty of Health and Medical Sciences, University of Adelaide, Adelaide, South Australia, Australia; Department of Gastroenterology, Southern Adelaide Local Health Network (SALHN) Flinders Medical Centre, Bedford Park South Australia, Australia; Adelaide Institute for Sleep Health, Flinders Health and Medical Research Institute, College of Medicine and Public Health, Flinders University, Bedford Park, South Australia, Australia; Adelaide Institute for Sleep Health, Flinders Health and Medical Research Institute, College of Medicine and Public Health, Flinders University, Bedford Park, South Australia, Australia; Department of Respiratory and Sleep Medicine, Southern Adelaide Local Health Network (SALHN) Flinders Medical Centre, Bedford Park, South Australia, Australia

**Keywords:** immune function, insomnia, sleep deprivation

## Abstract

**Study Objectives:**

Poor sleep-in people with inflammatory bowel disease (IBD) has been associated with worse quality of life, along with anxiety, depression, and fatigue. This meta-analysis aimed to determine the pooled prevalence of poor sleep-in IBD.

**Methods:**

Electronic databases were searched for publications from inception to November 1st 2021. Poor sleep was defined according to subjective sleep measures. A random effects model was used to determine the pooled prevalence of poor sleep-in people with IBD. Heterogeneity was investigated through subgroup analysis and meta-regression. Publication bias was assessed by funnel plot and Egger’s test.

**Results:**

519 Studies were screened with 36 studies included in the meta-analysis incorporating a total of 24 209 people with IBD. Pooled prevalence of poor sleep-in IBD was 56%, 95% CI (51–61%) with significant heterogeneity. The prevalence did not differ based on the definition of poor sleep. Meta-regression was significant for increased prevalence of poor sleep with increase in age and increased of prevalence of poor sleep with objective IBD activity but not subjective IBD activity, depression, or disease duration.

**Conclusions:**

Poor sleep is common in people with IBD. Further research is warranted to investigate if improving sleep quality in people with IBD will improve IBD activity and quality of life.

**Statement of Significance**

This meta-analysis of 36 studies, incorporating over 24 000 people with inflammatory bowel disease (IBD), showed that poor sleep quality is common in those with IBD and more frequent than reported estimates for fatigue and mental health conditions in people with IBD. Meta-regression showed that the differences in poor sleep between IBD populations related in part to IBD activity confirmed by objective measures. The presence of IBD related symptoms alone was not found to be significant.

## Introduction

Sleep is an important biologic function with increasing attention turning to its role in overall health. Abnormal sleep has been linked to poor health outcomes including cardiovascular disease [1], metabolic syndrome [2] and increased all-cause mortality in some studies [3], in addition to significant economic cost in the form of decreased productivity and increased health care utilization [4]. Sleep has been shown to regulate a number of gastrointestinal functions including gastrointestinal motility and secretion [5]. Sleep disruption has been associated with increased levels of inflammatory cytokines, such as IL-6, and TNF-α, that have been implicated in the pathogenesis of inflammatory bowel disease [6–8]. Poor sleep has been investigated in some chronic inflammatory diseases [9] and found to be prevalent in rheumatoid arthritis [10] and multiple sclerosis [11].

Inflammatory bowel disease (IBD) is an relapsing-remitting autoimmune disorder that results from a complex interaction between genetics and the environment [12]. IBD leads to a variety of debilitating symptoms such as diarrhea and abdominal pain. It is also associated with so called extra-intestinal manifestations, that include joint pain and skin rashes amongst others [13, 14]. Subjective assessment of the activity of IBD involves patient reported symptoms and utilizes validated scoring systems to ascertain the severity of IBD activity [15]. The reliability of subjective assessment of IBD activity is limited by the high prevalence of so called irritable bowel syndrome (IBS) like symptoms [16]. These IBS-like symptoms are often indistinguishable from the symptoms of active IBD and can occur in the absence of active IBD. Differentiating inactive IBD with IBS-like symptoms from active IBD requires the use of objective measures of IBD activity that directly confirm the presence of inflammation. These objective measures include endoscopic procedures such as colonoscopy, imaging such as magnetic resonance imaging and stool testing for markers of inflammation.

The relationship between IBD activity and sleep quality has been investigated previously with mixed results. A recent meta-analysis on the subject reached the conclusion that subjective sleep quality is worse in those with active IBD [17]. IBD related symptoms themselves, such as diarrhea and abdominal pain, may well disruptive sleep [18], however other studies suggest that endoscopically or histologically active IBD in the absence of any IBD symptoms may be sufficient to disrupt sleep [19, 20]. Extra-intestinal manifestations may also be important with a study suggesting those with enteropathic arthropathy were more likely to have poor sleep than those without [21]. Others suggest that psychosocial factors may be important [22], and in particular depression has been frequently associated with poor sleep [22–29] in an IBD population. Fatigue has also been associated with sleep quality [30–36] and is known to be highly prevalent in people with IBD [37].

Sleep may also be relevant to the development of IBD with data from the Nurses’ Health Study showing that sleep duration was associated with the risk of ulcerative colitis, but not Crohn’s disease [38]. Sleep quality may also have prognostic value in Crohn’s disease with sleep associations seen with increased likelihood of hospitalization and risk of relapse. The effect of IBD therapeutic agents on sleep has been investigated with a prospective study showing improvement in sleep following introduction of biologic therapy [27]—this of course paralleled an improvement in IBD activity. Others have not been able to demonstrate a relationship between the different IBD therapies and sleep quality [39].

In a recent meta-analysis subjective sleep quality was worse in those with IBD than controls [17]. This may be due to IBD associated symptoms, however there is some literature suggesting that those with inactive IBD also appear to have poor sleep [29, 33, 40, 41], although it is unclear if sleep quality in inactive IBD is worse than that of controls. Much of this data relates to subjective sleep quality with few studies incorporating objective sleep quality. Results from studies incorporating objective sleep quality are so far inconsistent noting a recent meta-analysis unable to establish an associated between objective sleep quality and IBD activity [17]. Furthermore, there was significant heterogeneity present in previous meta-analyses that is not well explained [17, 18].

This meta-analysis aimed to extend the work of the previous meta-analyses [17] by establishing the pooled prevalence of poor sleep-in IBD and exploring any heterogeneity that may be present. To the author’s knowledge there has been no previously published estimate of the prevalence of poor sleep-in IBD. An improved understanding of the burden of poor sleep-in IBD may lead to further investigation and interventional studies in this area that may result in improved quality of life for this population.

## Methods

This systematic review and meta-analysis was prospectively registered with the International Prospective Register of Ongoing Systematic Reviews [42]. It was performed according to the preferred reporting item for systematic reviews and meta-analyses (PRISMA) guidelines [43].

### Search strategy

Pubmed, MEDLINE, and PsychINFO were searched from inception to November 2021, including articles published in the English language using the following search string: (sleep OR circadian OR insomnia OR apnea) AND [(inflammatory bowel disease) OR (crohn’s disease) OR (ulcerative colitis) OR IBD OR crohn’s OR colitis)].

### Eligibility criteria

Studies were included if they met the following criteria: (1) cross-sectional, observational, case control, cohort or randomized controlled trial available (2) included a distinct population of people with inflammatory bowel disease (age ≥ 18 years old). Studies with control groups of a healthy population accepted. (3) Sleep quality assessment using a validated subjective patient reported measure of sleep.

Exclusion criteria included: (1) inappropriate study population such a pediatric or adolescent population. (2) Case report or review

### Study selection

The first author (AB) performed the literature review and two other authors (PS and JB) independently screened full texts against eligibility criteria, with disagreement resolved by discussion with involvement of another author (RM) when required.

### Data collection

Data collection was performed by AB. A pre-defined spreadsheet was used for data collection. Items collected for each study population included type of IBD, age, gender, study design, sample size, sleep assessment, outcome of study, disease activity in terms of subjective scores of disease activity or objective measures of disease activity, IBD disease duration, depression in terms of scores assessing depressive symptoms.

### Study quality assessment

Risk of bias in individual studies was assessed according to study design. Cross-sectional or observational studies were assessed according to modified Newcastle-Ottawa Scale. Cohort or case control studies were assessed according to Newcastle-Ottawa Scale [44].

Statistical analysis was performed using Stata SE 16 (StataCorp, College Station, TX, USA) and the ‘metaprop’ [45] command to estimate the pooled prevalence of poor sleep-in people with inflammatory bowel disease. Heterogeneity among studies was assessed using the I2 statistic with I2 > 50% considered to indicate substantial heterogeneity. A random effects model was used [45]. A Forest plot was performed to estimate individual and pooled effect sizes with associated 95% CI. Publication bias was assessed using funnel plots with significant visual asymmetry used to indicate publication bias. Egger’s test with *p* values less than .05 were considered to indicate significant publication bias. Trim-fill analysis was undertaken. In order to investigate sources of heterogeneity subgroup analysis and meta-regression were conducted.

## Results

**Figure 1.**
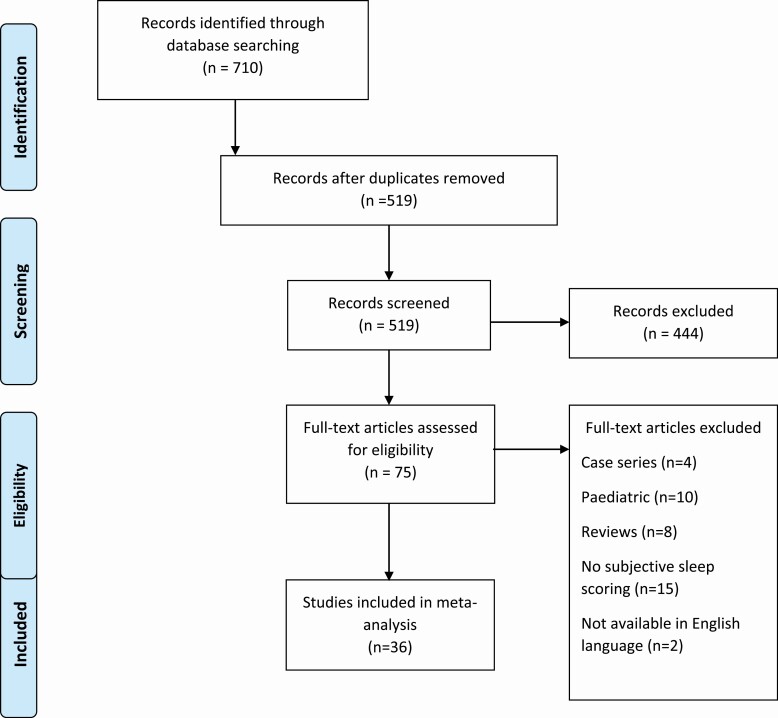
PRISMA flowchart—selection of studies and results of literature search for review and meta-analysis.

The literature search (see Figure 1) identified 519 records following removal of duplicates, which further reduced to 75 records following screening. Following exclusions 36 records were included in the meta-analysis incorporating 24 209 people with IBD.

### Study characteristics

Characteristics of included studies can be seen in Table 1 and further data in Supplementary Table 1. Publication dates ranged from 2011 to 2020. Most of the studies were single centre (*n* = 20), two were multi-centre, three recruited from an existing IBD registry, two recruited from a longitudinal cohort study, three used internet survey data, and two used data from a nationwide IBD cohort. The majority incorporated a cross-sectional design. No study included sample size calculations for prevalence estimates, and no study incorporated a population sampling regimen. Sample size ranged from 34 to 10 634 participants. The mean age of participants ranged from 25 to 45 years. The proportion of female participants ranged from 42 to 72%.

**Table 1.** Studies with people with inflammatory bowel disease with subjective sleep quality assessment

Study	Year	Country	Poor sleep definition	Study population	Population	Sample size	Percentage female (%)	Age	Number with Crohn’s disease	Proportion with poor sleep	Study summary
Abdalla et al. [49]	2017	USA	PROMIS-SD t score > 50	Patients within Crohn’s colitis foundation of America Partners Cohort	IBD	6309	71	44	3947	0.54	IBD-IBS diagnosis was associated with increased narcotic usage and poor sleep
Ali et al. [20]	2013	USA	PSQI > 5	Single centre—clinic	IBD	41	66	37	23	0.87	Clinically active IBD was associated with poor sleep
Ananthakrishnan et al. [29]	2013	USA	PROMIS-SD t score > 50	CCFA partners cohort	IBD	3173	72	44	2079	0.6	Sleep disturbance was associated with an increased risk of disease flares in Crohn’s disease but not ulcerative colitis
Ballou et al. [62]	2018	USA	PSQI > 5	Single centre—clinic	IBD	44	71	42	22	0.54	IBD patients at a tertiary clinic have poorer sleep than healthy controls
Bazin et al. [63]	2019	France	PSQI > 5	Single centre—clinic	Crohn’s disease	34	44	40	34	0.35	Sleep efficiency is lower in those active Crohn’s disease than in remission
Bucci et al. [64]	2018	Italy	PSQI > 5	Single centre—clinic	IBD	47	53	38	28	0.38	Bruxism was associated with pathological sleep
Calvo et al. [26]	2020	Spain	PSQI > 5	Single centre—clinic	IBD	102	43	45	51	0.54	Poor sleep quality is present in more than half of people with IBD
Chakradeo et al. [65]	2018	USA	PSQI > 5	Single centre—clinic	IBD	115	62	41		0.63	Later chronotype and markers of circadian misalignment were associated with IBD specific complications and lower quality of life
Chrobak et al. [31]	2018	Poland	PSQI > 5	Single centre—clinic	IBD	72	42	42	34	0.68	Chronotype preferences contribute to fatigue in IBD
Frigstad et al. [32]	2018	Norway	BSNQ	Multi-centre	IBD	405	49	40	227	0.19	Sleep and depressive symptoms were associated with total fatigue scores
Gîlc-Blanariu et al. [22]	2020	Romania	PSQI > 5	Single centre—clinic	IBD	110	47	44	34	0.75	Poor sleep is frequent in IBD and associated with psychological distress
Gingold-Belfer et al. [66]	2014	Israel	PSQI > 5	single centre—clinic	Crohn’s disease	108	47	40	108	0.37	Poor sleep is associated with active Crohn’s disease but not inactive disease
Graff et al. [33]	2011	USA	PSQI > 5	Manitoba IBD cohort	IBD	318	60	43	160	0.49	Poor sleep is prevalent in those with active IBD but also in those with inactive IBD
Habibi et al. [67]	2019	Iran	PSQI > 5	Single centre—clinic	IBD	68	63	38	24	0.32	Poor sleep is prevalent in those with IBD including those in remission
Hashash et al. [34]	2016	USA	PSQI > 5	Single centre—registry	IBD	685	53	44	418	0.54	Fatigue was associated wit poor sleep and psychopathology
Hood et al. [24]	2018	USA	PSQI > 5	Multi-centre—clinic	IBD	47			0	0.59	Poor sleep is prevalent in ulcerative colitis and related to depression
IsHak et al. [48]	2017	USA	PROMIS-SD t score > 50	Single centre—clinic	IBD	110	43	42	62	0.6	Patient’s with Crohn’s disease demonstrated worse impairments in quality of life and function than those with ulcerative colitis
Iskandar et al. [68]	2020	USA	PSQI > 5	Single centre—clinic	Crohn’s disease	61		32	61	0.57	Crohn’s disease patients reported more disturbed sleep than controls but this was not confirmed with objective measures
Kani et al. [69]	2019	Turkey	PSQI > 5	Single centre—clinic	IBD	136	58	39	72	0.59	Dream anxiety may lead to sleep disturbance in patients with IBD
Kappelman et al. [47]	2014	USA	PROMIS-SD t score > 50	internet cohort multi-centre	IBD	10634	71	44	6689	0.58	Health outcomes measures differ between patients with IBD and the general population
Keskin et al. [70]	2020	Turkey	PSQI > 5	Single centre—clinic	IBD	89	56	37	41	0.51	IBD risk factor for sleep disturbance with eveningness more common than in controls
Lee et al. [39]	2018	USA	PSQI > 5	Single centre—clinic	IBD	56	66	45	39	0.82	Treatment with immuno-modulators or biologics does not appear to improve sleep quality
Marinelli et al. [25]	2020	Italy	PSQI > 5	Single centre—clinic	IBD	166	47	44	87	0.67	Sleep quality was not associated with IBD activity but with mood, disability and quality of life
Michalopoulos et al. [19]	2018	Greece	PSQI > 5	Single centre—clinic	IBD	90	46	40	54	0.45	In IBD in clinical remission endoscopic findings was associated with poor sleep
Schindlbeck et al. [36]	2016	Germany	PSQI > 5	Single centre—clinic	IBD	43	72	47	30	0.61	Restless leg syndrome in inflammatory disease with associated with worse quality of life
Sobolewska-Włodarczyk et al. [71]	2018	Poland	PSQI > 5	Single centre—clinic	IBD	65	43	40	30	0.69	Poor sleep-in IBD related to IBD activity
Sobolewska-Włodarczyk et al. [72]	2020	Poland	PSQI > 5	Single centre—clinic	IBD	65	47	40	30	0.57	Specific adipokine profiles are associated with circadian rhythms
Sochal et al. [23]	2020	Poland	PSQI > 5	Single centre—clinic	IBD	133	55	37	68	0.43	Poor sleep-in IBD is common and related to mood
Sofia et al. [73]	2019	USA	PSQI>5	Single centre—clinic	IBD	92	62	43	92	0.77	Poor sleep is common in Crohn’s disease and associated with adverse outcomes
Stevens et al. [27]	2016	USA	PROMIS-SD t score > 50	Single centre—registry	IBD	160	48	35	94	0.44	Vedolizumab and anti-TNF biologics were associated with improvement in sleep quality
Takahara et al. [74]	2016	Japan	PSQI > 5	Single centre—clinic	IBD	80	42	42	34	0.4	Restless leg syndrome occurs frequently in Japanese patients with IBD
Uemura et al. [75]	2016	Japan	PSQI > 5.5	Single centre—clinic	IBD	136	44	42	48	0.44	Sleep disturbance common in Japanese IBD patients and associated with poor quality of life
van Langenberg et al. [76]	2017	Australia	PSQI > 5	Single centre—clinic	IBD	49	58	44	49	0.63	Crohn’s disease patients demonstrated subtle cognitive impairment
Wilson et al. [28]	2014	USA	PROMIS-SD t score > 50	single centre—registry	IBD	131	55	25	78	0.44	High CRP associated with poor sleep irrespective of night-time disruptions
Zargar et al. [77]	2019	Iran	PSQI > 5	Single centre—clinic	IBD	115	49	38	30	0.51	IBS may worsen sleep disturbance in IBD
Zhang et al. [21]	2020	China	PSQI > 5	Single centre—clinic	IBD	120	50	36	39	0.99	Sleep quality in those with peripheral arthropathy and IBD was worse than those without

Characteristics of studies included in the meta-analysis of poor sleep prevalence. See Supplementary Table 1 for further details.

USA, United States of America; IBD, inflammatory bowel disease; PSQI, Pittsburgh Sleep Quality Index; BSNQ, Basic Nordic Sleep Questionnaire—first question used.

### Measurement of sleep quality

The Pittsburgh Sleep Quality Index (PSQI) was reported in the majority of included studies (*n* = 29) (see Table 2).The PSQI is a validated measure to assess perceived sleep quality [46]. The index consists of subscales on sleep duration, sleep disturbance, sleep latency, daytime dysfunction, sleep efficiency, overall sleep quality and medications for sleep. The score ranges from 0 to 21, with a PSQI > 5 considered to represent poor sleep quality. PSQI sub-scores were reported in seven studies and consequently this was not investigated further.

**Table 2.** Meta-regression performed for prevalence of poor sleep

	Number of studies	Coefficient	Standard error	*P* value	Residual heterogeneity (I2%)
Crohn’s disease	31	0.0147	0.10	.88	96.8
Age	36	0.017	0.006	.005	95.6
Female gender	33	0.002	0.002	.28	96.5
IBD disease duration	18	0.005	0.007	.54	97.6
Objective IBD activity	8	0.64	0.17	.001	30.5
Subjective IBD activity	25	0.013	0.21	.95	98.0
Depression	15	0.13	0.17	.43	95.6

IBD, inflammatory bowel disease.

The Patient Reported Outcomes Measurement Information Systems sleep disturbance (PROMIS-SD) questionnaire was used by six studies [27–29, 47–49]. The PROMIS-SD questionnaire was developed by the National Institute of Health [50]. The PROMIS-SD has comparable performance to the PSQI in identifying poor sleep [51]. A PROMIS-SD *t* score over 50 is referred to as poor sleep. A single study [32] used the Basic Nordic Sleep Questionnaire [52] (BNSQ), utilizing the first dimension of the BNSQ and a score above 3 considered significant.

### Prevalence of poor sleep-in IBD

**Figure 2.**
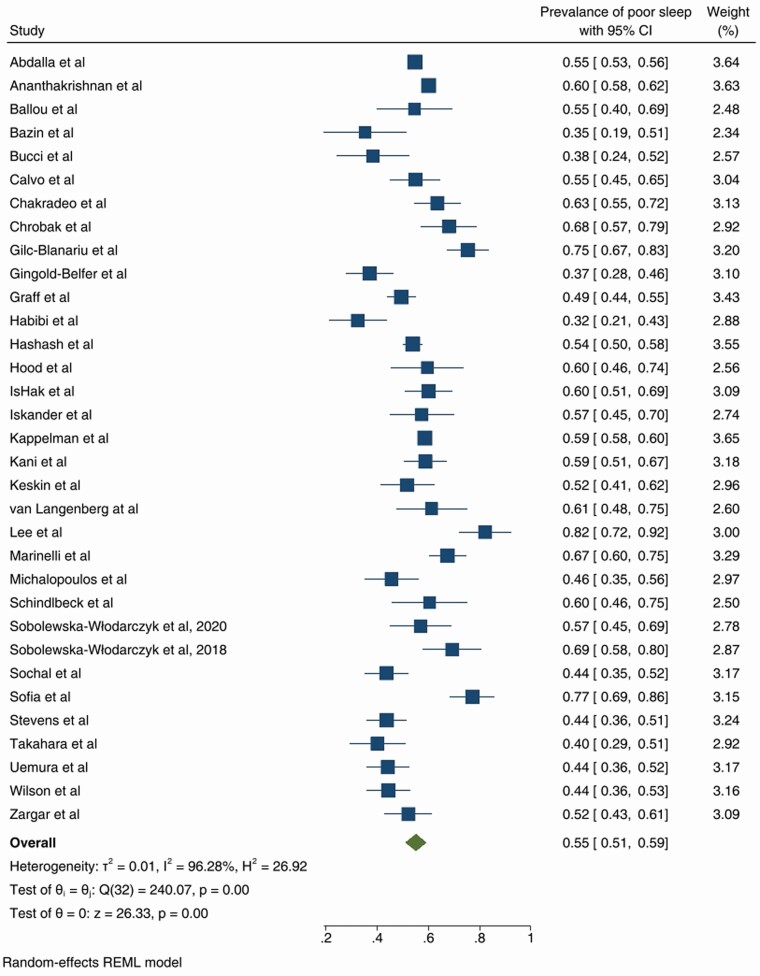
Forest plot of the prevalence of poor sleep-in those with inflammatory bowel disease.

The prevalence of poor sleep varied from 32 to 99%, with random effects model derived pooled prevalence of 55%, 95% CI (51–59) with substantial heterogeneity (Forest plot in Figure 2), outliers were removed [21, 32]. Funnel plot was symmetric (Supplementary Figure 1) and Egger’s test not significant (*p* = .49). The Trim-fill method did not suggest any additional studies.

### Subgroup analysis

Subgroup analysis was performed for definition of poor sleep, study of origin and publication date. There was no difference in the prevalence of poor sleep by definition of poor sleep (PSQI or PROMIS-SD sleep, *p* = .75). Most studies were from the United States of America (*n* = 15), followed by Europe (*n* = 12), and others including Australia, Japan, Turkey and Iran. The pooled prevalence was similar between Europe (56% [49–63]), and the USA (58% [53–64], both of which were significantly different to other (Australia, Japan, Turkey, Iran) (44% [36–52]) (*p* = .01)(see Supplementary Figure 2). Publication date subgroups were considered from 2011 to 2016 (*n* = 10), and 2017 to 2020 (*n* = 23). The prevalence of poor sleep was higher in the 2017 to 2020 subgroup (*p* = .03, 58% [53–63] v 50% [44–55]). However, altering the publication date subgroups by a single year resulting in no effect seen, discounting the above result.

### Meta-regression

Meta-regression was performed for demographics and IBD related data (see Table 2 and supplementary Table 3). Age was significant (*p* = .005) with increasing proportion of poor sleep associated with increase in age, however residual heterogeneity remaining significant (I2 95.6%). Meta-regression was not significant for gender (*p* = .28), IBD type (*p* = .88), and IBD disease duration (*p* = .54).

### IBD activity

IBD activity was reported in 25 studies (*n* = 23 229) in the form of subjective disease activity scores such as the Harvey Bradshaw Index [15] or the Crohn’s disease activity index [53] (see Supplementary Table 2). The meta-regression incorporated the number of people with active IBD as per these subjective disease activity scores. Meta-regression for subjective disease activity was not significant (*p* = .95). Objective IBD activity was reported in eight studies (*n* = 1931), with objective measures including C-reactive protein, fecal calprotectin, endoscopic findings and histology (see Supplementary Table 3). On meta-regression objective IBD activity was significant (*p* = .001), increasing proportion of poor sleep was associated with increase in objective disease activity. Residual heterogeneity was I2 30.5%, this is as compared to heterogeneity for these eight studies at I2 of 82.6%, suggesting that objective disease activity may explain much of the inter-study heterogeneity.

### Depression

Assessment of depression was performed in 15 studies (*n* = 10 744) (see Supplementary Table 4). Eight of these studies reported a significant association between poor sleep quality and depression [22–29]. Scoring systems included the Hospital Anxiety and Depression Scale [54] (*n* = 6), PROMIS [55] depression score (*n* = 4), Beck’s Depression Inventory II [56] (*n* = 3), depressive symptoms (*n* = 1) and depression under treatment (*n* = 1). On meta-regression depression was not significant (*p* = .43).

## Discussion

This is the largest and only meta-analysis to date providing prevalence estimates for poor sleep-in IBD. The pooled prevalence for poor sleep-in IBD was high (55%), eclipsing that reported in a recent meta-analysis of fatigue (47%) [37], and of symptoms of anxiety (32%) and depression (25%) [4]. The prevalence of poor sleep reported here is of a higher magnitude than the prevalence of sleep disorder in IBS with a recent meta-analysis reporting a pooled prevalence of 37.6% [57]. This highlights the importance of poor sleep-in IBD and suggests further resources should be allocated to investigate this area.

Sources of heterogeneity in the prevalence estimate of poor sleep included age, geographic location, and objective disease activity. Age-related sleep changes have been well described with decreasing sleep quality accepted [58], with a similar association between age and sleep quality seen in a rheumatoid arthritis population [59, 60]. It was considered that the significance of age may also relate to IBD disease duration, however this was not significant on meta-regression.

Objective IBD activity did vary between studies and was a significant source of heterogeneity. A recent meta-analysis was unable to elicit a significant relationship between objective IBD activity and sleep [17], which may in part be due to the small number of studies utilizing objective measures of IBD activity. It did however find that subjective IBD activity was associated with poor sleep quality—a finding not replicated here despite the variance between different studies. This suggests that the underlying inflammatory response may be more significant than the associated symptoms, consistent with studies associated histology activity and endoscopic activity in the absence of symptoms with poor sleep [19, 20]. IBD activity is likely not the only driver of poor sleep quality with several studies reporting frequent poor sleep-in those with inactive disease [29, 33, 40, 41].

Depression was not a significant source of heterogeneity despite varying between studies and despite several positive findings in the literature [22–29]. Low physical activity [31, 61] and the presence of extra-intestinal IBD manifestations [21] have also been associated with poor sleep, unfortunately these were reported a minority of studies making further investigation impractical.

### Limitations

As a result of the paucity of studies incorporating objective sleep assessments, we used a definition of poor sleep based on self-reported sleep quality. There is a suggestion in some studies [25] that people with IBD will report significantly worse sleep than can be substantiated objectively, and consequently the true prevalence of poor sleep may be lower. This supports the need for objective sleep assessments in people with IBD. Other limitations include most studies being single centre, although results were similar to multi-centre or nationwide studies. Although we note that prevalence from nationwide studies was similar to other single centre studies. No study incorporated sample size calculations or included a rigorous sampling approach. There was a general lack of demographic data reported in studies, such as race, that may have a significant influence on the prevalence of poor sleep-in this population. The differentiation between gender and sex was not well defined in most studies. Finally, a single reviewer was responsible for data collection.

### Future work

Further work should consider studies incorporating objective disease and sleep quality measurements to understand the relationship and type of sleep disorders in this population. There are few interventional studies in this area, with a need to establish if the potential benefit of improving sleep-in people with IBD would extend beyond quality of life to incorporate IBD related outcomes such as IBD activity, and surgery. There is also the lack of simple IBD specific screening tool for use in IBD clinic to identify those with poor sleep who would benefit from referral onto a sleep physician.

## Conclusions

This meta-analysis has demonstrated that the prevalence of poor sleep-in IBD is significant, although there was substantial heterogeneity between studies. Meta-regression demonstrated that age and objective IBD activity were significant, with subjective IBD activity not significant. Objective IBD activity explained most of the heterogeneity between studies. Further research is required in this area to establish the relationship between IBD activity and sleep quality and to consider sleep targeted interventions in an IBD population.

## Supplementary Material

zpac025_suppl_Supplementary_Material

## Data Availability

*The data underlying this article are available in* the Harvard Dataverse Digital Repository at https://doi.org/10.7910/DVN/FVLPYA
