# Supplementary material for: A systematic review and meta-analysis of the prevalence of poor sleep in inflammatory bowel disease
Source: Sleep Adv. 2022 Aug 26;3(1):zpac025. doi: 10.1093/sleepadvances/zpac025 (PMC10104416; doi:10.1093/sleepadvances/zpac025)

A systematic review and meta-analysis of the prevalence of poor sleep in inflammatory bowel disease

Dr Alex Barnes MD MPH FRACP^1,2^

Dr Réme Mountifield MBBS PhD FRACP ^1,2^

Dr Justin Baker MD^1,2^

Dr Paul Spizzo MBBS PhD FRACP ^1^

Dr Peter Bampton MBBS MD FRACP AGAF^2^

Professor Jane M Andrews MBBS PhD FRACP AGAF^4,5^

Professor Robert J. Fraser MBBS PhD FRACP AGAF^1,2^

A/Professor Sutapa Mukherjee MBBS PhD FRACP^2,3^

1. Department of Gastroenterology, Southern Adelaide Local Health Network (SALHN) Flinders Medical Centre, Bedford Park South Australia, Australia

2. Adelaide Institute for Sleep Health, Flinders Health and Medical Research Institute, College of Medicine and Public Health, Flinders University, Bedford Park, South Australia, Australia

3. Department of Respiratory and Sleep Medicine, Southern Adelaide Local Health Network (SALHN) Flinders Medical Centre, Bedford Park, South Australia, Australia

4. Inflammatory Bowel Disease Service, Department of Gastroenterology and Hepatology, (CAHLN) Royal Adelaide Hospital, Adelaide, South Australia, Australia

5. School of Medicine, Faculty of Health & Medical Sciences, University of Adelaide, Adelaide, South Australia, Australia

Corresponding author:

Dr Alex Barnes

Flinders Medical Centre, Flinders Drive

Bedford Park, SA 5042, Australia

Phone: 614882045511, Fax: 614882045555

Email: barn0255@flinders.edu.au

**Author contributions**

Alex Barnes: responsible for study concept and design, data acquisition, analysis and data interpretation, drafting of manuscript, critical revision of the manuscript.

Paul Spizzo: responsible for data acquisition, data interpretation, drafting of manuscript, critical revision of the manuscript.

Justin Baker: responsible for data acquisition and critical revision of the manuscript.

Peter Bampton: responsible for critical revision of the manuscript.

Jane Andrews: responsible for study conception

Robert J Fraser: responsible for study conception

Réme Mountifield: responsible for critical revision of the manuscript.

Sutapa Mukherjee: responsible for study concept and design, critical revision of the manuscript

**Conflicts of interest include** speakers fees, and Ad Boards from : Abbott, AbbVie, Allergan, Anatara, AstraZeneca, Bayer, BMS 2020, Celegene, Celltrion, Falk, Ferring, Gilead, Hospira, Immuninc, ImmunsanT, Janssen, MSD, Nestle, Novartis, Progenity, Pfizer, Sandoz, Shire, Takeda, Vifor, RAH research Fund, The Hospital Research Fund 2020-2022, The Helmsley Trust 2020-2023

**Data availability statement**

The data underlying this article are available in the Harvard Dataverse Digital Repository at https://doi.org/10.7910/DVN/FVLPYA

Supplemental figure 1: Funnel plot of meta-analysis for prevalence of poor sleep in those with inflammatory bowel disease.

Supplementary figure 2: Subgroup analysis of prevalence of poor sleep by geographic region

Supplementary table 1: Study quality scored according to the Newcastle-Ottawa scale with sub-scores and associated study quality detailed.

Supplementary table 2: Study characteristics including subjective IBD activity and IBD disease duration.

Supplementary table 3: Characteristics of studies incorporating objective IBD activity.

Supplementary table 4: Characteristics of studies incorporating assessment of depression.

Supplementary table 1: Study quality scored according to the Newcastle-Ottawa scale with sub-scores and associated study quality detailed.

| Study | Year | Selection | Comparability | Outcome | Study quality |
| --- | --- | --- | --- | --- | --- |
| Abdalla et al | 2017 | 3 | 1 | 2 | Good |
| Ali et al | 2013 | 3 | 1 | 3 | Good |
| Ananthakrishnan et al | 2013 | 3 | 1 | 2 | Good |
| Ballou et al | 2018 | 2 | 1 | 2 | Fair |
| Bazin et al | 2019 | 3 | 1 | 3 | Good |
| Bucci et al | 2018 | 2 | 1 | 2 | Fair |
| Calvo et al | 2020 | 3 | 2 | 3 | Good |
| Chakradeo et al | 2018 | 4 | 2 | 2 | Fair |
| Chrobak et al | 2018 | 2 | 2 | 2 | Fair |
| Frigstad et al | 2018 | 3 | 2 | 2 | Good |
| Gîlc-Blanariu et al | 2020 | 4 | 1 | 3 | Good |
| Gingold-Belfer et al | 2014 | 3 | 1 | 3 | Good |
| Graff et al | 2011 | 3 | 2 | 3 | Good |
| Habibi et al | 2019 | 4 | 2 | 3 | Good |
| Hashash et al | 2016 | 2 | 2 | 3 | Fair |
| Hood et al | 2018 | 2 | 1 | 3 | Fair |
| IsHak et al | 2017 | 2 | 1 | 2 | Fair |
| Iskandar et al | 2020 | 2 | 2 | 3 | Fair |
| Kani et al | 2019 | 2 | 2 | 1 | Fair |
| Kappelman et al | 2014 | 3 | 2 | 2 | Good |
| Keskin et al | 2020 | 2 | 1 | 2 | Fair |
| Lee et al | 2018 | 3 | 2 | 2 | Fair |
| Marinelli et al | 2020 | 2 | 2 | 2 | Fair |
| Michalopoulos et al | 2018 | 2 | 2 | 3 | Fair |
| Schindlbeck et al | 2016 | 4 | 2 | 3 | Good |
| Sobolewska-Włodarczyk et al | 2018 | 3 | 1 | 3 | Good |
| Sobolewska-Włodarczyk et al | 2020 | 3 | 1 | 2 | Fair |
| Sochal et al | 2020 | 3 | 1 | 3 | Good |
| Sofia et al | 2019 | 3 | 1 | 2 | Good |
| Stevens et al | 2016 | 4 | 2 | 3 | Good |
| Takahara et al | 2016 | 3 | 2 | 2 | Good |
| Uemura et al | 2016 | 3 | 2 | 3 | Good |
| van Langenberg at al | 2017 | 2 | 2 | 2 | Fair |
| Wilson et al | 2014 | 3 | 2 | 2 | Good |
| Zargar et al | 2019 | 4 | 2 | 2 | Good |
| Zhang et al | 2020 | 2 | 2 | 2 | Fair |

Supplementary table 2: Study characteristics including subjective IBD activity and IBD disease duration.
HADS-D Hospital anxiety and depression scale depression sub score; BDI-II Beck’s Depression Inventory II; IBD – inflammatory bowel disease; PSQI – Pittsburgh Sleep Quality Index; CRP – C-reactive protein

| Study | Year | Sample size | Number with Crohn's disease | Disease duration (mean, years) | Proportion with poor sleep | Proportion with active IBD |
| --- | --- | --- | --- | --- | --- | --- |
| Abdalla et al | 2017 | 6309 | 3947 |  | 0.54 | 0.42 |
| Ali et al | 2013 | 41 | 23 |  | 0.87 | 0.49 |
| Ananthakrishnan et al | 2013 | 3173 | 2079 | 7.6 | 0.6 | 0.43 |
| Ballou et al | 2018 | 44 | 22 |  | 0.54 |  |
| Bazin et al | 2019 | 34 | 34 |  | 0.35 | 0.41 |
| Bucci et al | 2018 | 47 | 28 | 7 | 0.38 |  |
| Calvo et al | 2020 | 102 | 51 | 8 | 0.54 |  |
| Chakradeo et al | 2018 | 115 |  | 7 | 0.63 |  |
| Chrobak et al | 2018 | 72 | 34 |  | 0.68 |  |
| Frigstad et al | 2018 | 405 | 227 | 11.5 | 0.19 | 0.38 |
| Gîlca-Blanariu et al | 2020 | 110 | 34 |  | 0.75 | 0.49 |
| Gingold-Belfer et al | 2014 | 108 | 108 | 15 | 0.37 | 0.34 |
| Graff et al | 2011 | 318 | 160 | 15 | 0.49 | 0.46 |
| Habibi et al | 2019 | 68 | 24 |  | 0.32 | 0.75 |
| Hashash et al | 2016 | 685 | 418 |  | 0.54 | 0.21 |
| Hood et al | 2018 | 47 | 0 |  | 0.59 |  |
| Ishak et al | 2017 | 110 | 62 |  | 0.6 |  |
| Iskandar et al | 2020 | 61 | 61 |  | 0.57 | 0.55 |
| Kani et al | 2019 | 136 | 72 |  | 0.59 | 0.17 |
| Kappelman et al | 2014 | 10634 | 6689 | 7.5 | 0.58 | 0.5 |
| Keskin et al | 2020 | 89 | 41 | 8.7 | 0.51 |  |
| van Langenberg at al | 2017 | 49 | 49 | 10.1 | 0.63 | 0.42 |
| Lee et al | 2018 | 56 | 39 | 6.4 | 0.82 | 0.46 |
| Marinelli et al | 2020 | 166 | 87 |  | 0.67 | 0.47 |
| Michalopoulos et al | 2018 | 90 | 54 |  | 0.45 | 0.43 |
| Schindlbeck et al | 2016 | 43 | 30 | 5.7 | 0.61 |  |
| Sobolewska-Włodarczyk et al | 2018 | 65 | 30 | 14.9 | 0.69 | 0.78 |
| Sobolewska-Włodarczyk et al | 2020 | 65 | 30 | 3 | 0.57 | 0.8 |
| Sochal et al | 2020 | 133 | 68 |  | 0.43 | 0.62 |
| Sofia et al | 2019 | 92 | 92 | 5.5 | 0.77 | 0.15 |
| Stevens et al | 2016 | 160 | 94 | 5.8 | 0.44 |  |
| Takahara et al | 2016 | 80 | 34 | 13 | 0.4 |  |
| Uemura et al | 2016 | 136 | 48 |  | 0.44 | 0.16 |
| Wilson et al | 2014 | 131 | 78 | 11.3 | 0.44 | 0.42 |
| Zargar et al | 2019 | 115 | 30 |  | 0.51 |  |
| Zhang et al | 2020 | 120 | 39 |  | 0.99 | 0.35 |

Supplementary table 3: Characteristics of studies incorporating objective IBD activity.
IBD – inflammatory bowel disease; CRP – C-reactive protein

| Study | Year | Proportion with objectively active IBD | Definition of objective IBD activity | Proportion with poor sleep |
| --- | --- | --- | --- | --- |
| Ali et al | 2013 | 0.71 | histology | 0.87 |
| Frigstad et al | 2018 | 0.31 | CRP > 5 | 0.19 |
| Graff et al | 2011 | 0.23 | CRP > 8 | 0.49 |
| Hashash et al | 2016 | 0.216 | CRP > 7 | 0.54 |
| Kani et al | 2019 | 0.37 | CRP >7 | 0.59 |
| van Langenberg at al | 2017 | 0.27 | CRP > 5 | 0.63 |
| Marinelli et al | 2020 | 0.47 | Calprotectin > 250 | 0.67 |
| Wilson et al | 2014 | 0.19 | CRP > 8 | 0.42 |

Supplementary table 4: Characteristics of studies incorporating assessment of depression.
HADS-D Hospital anxiety and depression scale depression sub score; BDI-II Beck’s Depression Inventory II.

| Study | Year | Proportion with poor sleep | Proportion with active IBD | Proportion with depression | Depression score |
| --- | --- | --- | --- | --- | --- |
| Abdalla et al | 2017 | 0.54 | 0.42 | 0.01 | BDI-II |
| Ali et al | 2013 | 0.87 | 0.49 | 0.074 | HADS-D |
| Ananthakrishnan et al | 2013 | 0.6 | 0.43 | 0.089 | Depression under treatment |
| Ballou et al | 2018 | 0.54 |  | 0.091 | BDI-II |
| Bazin et al | 2019 | 0.35 | 0.41 | 0.11 | HADS-D |
| Bucci et al | 2018 | 0.38 |  | 0.2 | HADS-D |
| Calvo et al | 2020 | 0.54 |  | 0.24 | PROMIS-SD-depression |
| Chakradeo et al | 2018 | 0.63 |  | 0.25 | BDI-II |
| Chrobak et al | 2018 | 0.68 |  | 0.5 | HADS-D |
| Frigstad et al | 2018 | 0.19 | 0.38 | 0.5 | HADS-D |
| Gîlca-Blanariu et al | 2020 | 0.75 | 0.49 | 0.5 | PROMIS-SD-depression |
| Gingold-Belfer et al | 2014 | 0.37 | 0.34 | 0.51 | PROMIS-SD-depression |
| Graff et al | 2011 | 0.49 | 0.46 | 0.51 | Depressive symptoms |
| Habibi et al | 2019 | 0.32 | 0.75 | 0.52 | HADS-D |
| Hashash et al | 2016 | 0.54 | 0.21 | 0.55 | PROMIS-SD-depression |

Supplemental Figure 1: Funnel plot of meta-analysis for prevalence of poor sleep in those with inflammatory bowel disease.


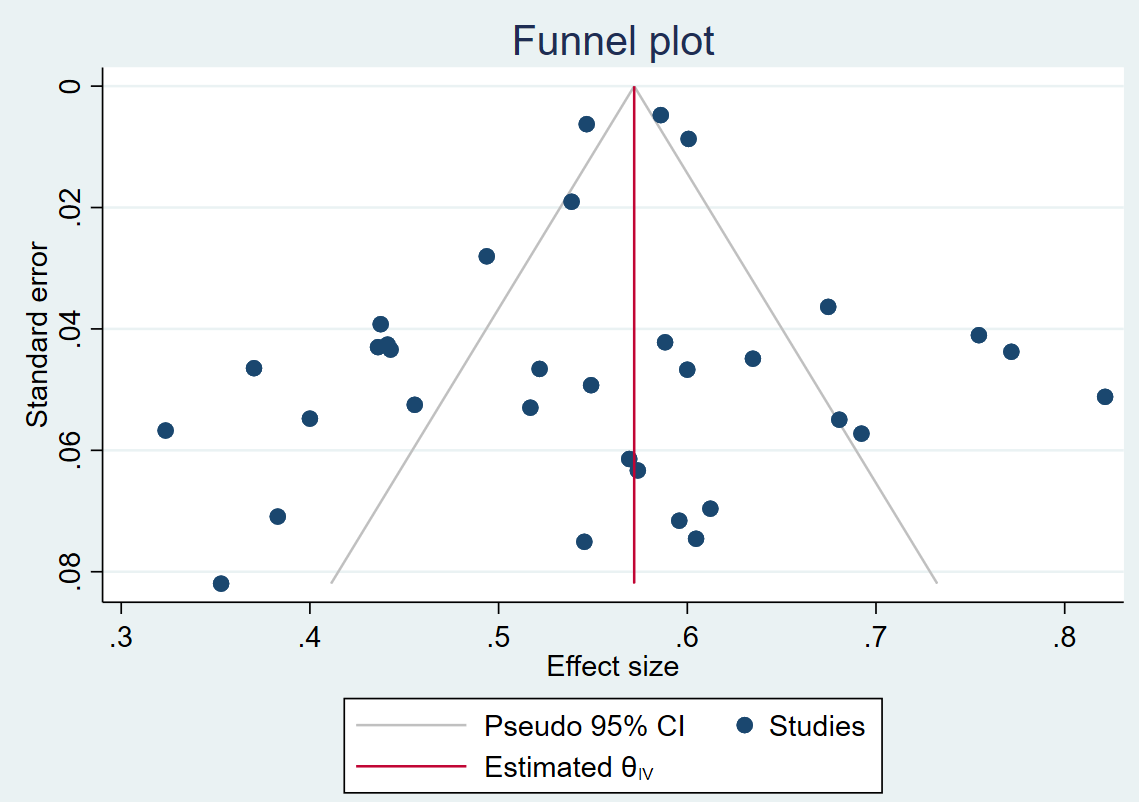


Supplementary figure 2: Subgroup analysis of prevalence of poor sleep by geographic region


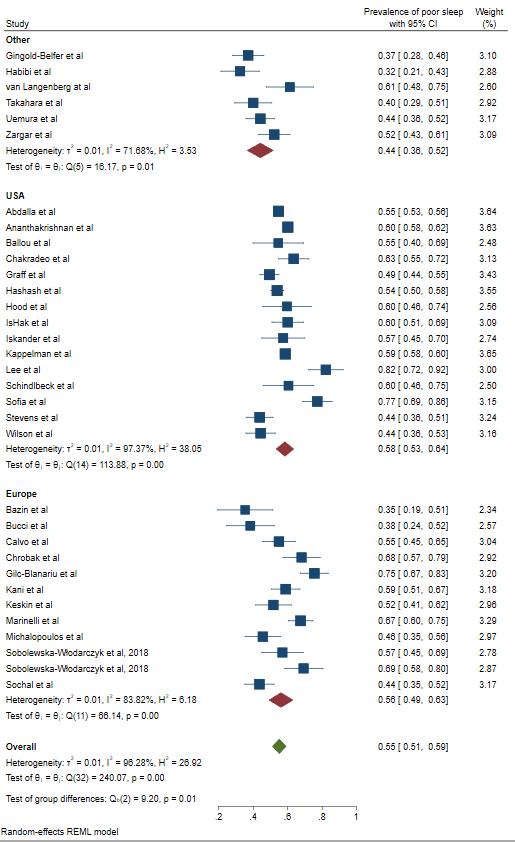

Supplement: zpac025_suppl_Supplementary_Material [file zpac025_suppl_supplementary_material.docx]
